# Combining Synthetic Images and Deep Active Learning: Data-Efficient Training of an Industrial Object Detection Model

**DOI:** 10.3390/jimaging10010016

**Published:** 2024-01-06

**Authors:** Leon Eversberg, Jens Lambrecht

**Affiliations:** Industry Grade Networks and Clouds, Faculty IV Electrical Engineering and Computer Science, Technische Universität Berlin, Straße des 17. Juni 135, 10623 Berlin, Germany; lambrecht@tu-berlin.de

**Keywords:** active learning, computer vision, data efficiency, deep active learning, deep learning, image synthesis, industrial application, object detection, synthetic images, turbine blade

## Abstract

Generating synthetic data is a promising solution to the challenge of limited training data for industrial deep learning applications. However, training on synthetic data and testing on real-world data creates a sim-to-real domain gap. Research has shown that the combination of synthetic and real images leads to better results than those that are generated using only one source of data. In this work, the generation of synthetic training images via physics-based rendering is combined with deep active learning for an industrial object detection task to iteratively improve model performance over time. Our experimental results show that synthetic images improve model performance, especially at the beginning of the model’s life cycle with limited training data. Furthermore, our implemented hybrid query strategy selects diverse and informative new training images in each active learning cycle, which outperforms random sampling. In conclusion, this work presents a workflow to train and iteratively improve object detection models with a small number of real-world images, leading to data-efficient and cost-effective computer vision models.

## 1. Introduction

Deep learning has become a key technology for solving real-world industrial problems using artificial intelligence. However, deep learning models often require large-scale datasets to achieve adequate performance. Limited data remains one of the major challenges for industrial applications of deep learning [1]. As a solution for computer vision tasks, synthetic images can be generated and used as training data. Generating synthetic images has many advantages compared to collecting and manually annotating real-world images. Synthetic images are fast and cheap to generate. They can be used to balance out real-world dataset biases [2]. Furthermore, they can be used in situations where there are privacy concerns surrounding the usage of real-world images [3]. Additionally, they have pixel-perfect annotations without the potential for human error [4].

However, using synthetic images to train computer vision models and then testing them on real-world images creates a domain gap that continues to be a challenge in this field of research [5]. Research has shown that the combination of synthetic and real images outperforms the use of a single data source [6,7,8,9,10,11]. But how can real-world training images be efficiently selected for combination with the generated synthetic images? In this work, we propose to solve this problem with strategies from the field of active learning (AL). AL uses the current machine learning model to efficiently select data for the next iteration of training.

This paper builds upon our previous work to generate training images via physics-based rendering for industrial object detection (OD) tasks [11] and makes the following new contributions:

- A workflow is presented to efficiently train industrial object detection models by automatically generating synthetic training images based on 3D models and then using deep active learning to iteratively improve the model with reduced annotation cost.
- Different deep active learning query strategies are investigated on a collected industrial dataset for a real-world object detection use case.
- Multiple deep active learning cycles are compared to a single cycle with an equivalent amount of manually labeled training images.

The remainder of this paper is structured as follows: Section 2 provides a summary of prior work on synthetic images and deep active learning for object detection tasks. In Section 3, the methodology of this paper is presented. Our results for synthetic versus real images and different deep active learning (DAL) query strategies are presented in Section 4. Lastly, Section 5 outlines the limitations of our study and summarizes our primary findings.

## 2. Related Works

### 2.1. Using Synthetic Images to Train Computer Vision Models

Generating synthetic training data is a promising solution to the data-hungry nature of modern deep learning models. However, training models on a source domain of synthetic images and testing them on a target domain of real images leads to a domain gap, which remains one of the biggest challenges in this field [12]. In order to overcome the domain gap, different approaches have been used. A simple strategy is to copy objects from real images and then paste them onto random background images to create new images [13,14]. For industrial applications, available 3D models can be used to train object detection models [15]. Domain randomization is an approach where training images are randomized to such an extent that the trained model is supposed to see real images as just another variation of the synthetic training data [5,16,17]. The concept of photorealism is another approach, where the goal is to create highly realistic images using physics-based rendering [8,18,19]. Physics-based rendering uses the ray-tracing algorithm to follow the path of light rays through the virtual scene as they bounce off objects in the scene [20]. Domain adaptation is a third approach to bridging the domain gap. This technique attempts to make the source domain and the target domain as similar as possible through image transformations. Synthetic images can be transformed closer to the target domain using generative adversarial networks [21,22,23]. Alternatively, image filters can be used to transform both source and target images to an intermediate domain [24,25].

### 2.2. Deep Active Learning

**Figure 1:**
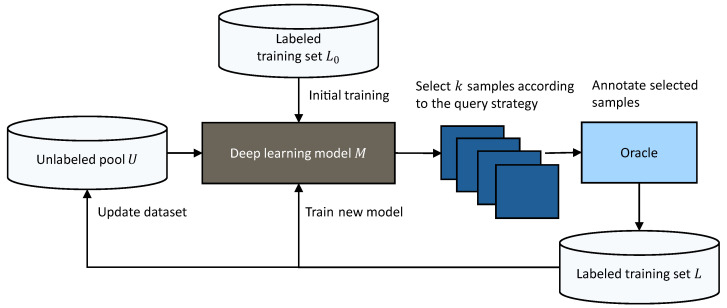
Deep active learning cycle. The large unlabeled pool *U* is used as input for the current deep learning model. Based on the extracted features, a query strategy selects a batch of *k* optimal samples for annotation, which can then be used in the next training iteration. Figure based on [27].

AL is a subfield of machine learning that attempts to maximize the performance of a machine learning model with the least amount of annotated data. The key idea behind AL is that the model selects the data from which it learns [26]. In traditional AL, most algorithms query only one sample at a time, which is inefficient for modern deep learning. Therefore, DAL uses a batch-based query strategy to select the *k* most useful samples from a large unlabeled pool of data *U* for annotation to reduce labeling cost while maintaining performance [27]. To select optimal query samples, unlabeled data are fed into the model to generate features. Given these features, a query strategy attempts to find an optimal batch of samples. The selected *k* samples are annotated by the oracle, e.g., a human annotator, and are then added to the labeled training set *L*. Given the updated labeled training set, a new model can be trained. This DAL cycle is depicted in Figure 1. The first iteration of the DAL cycle requires an initial model to be trained on the initial labeled training set $L_{0}$ .

Query strategies can be classified into the following three categories: uncertainty-based query strategies, diversity-based query strategies, and hybrid strategies that combine uncertainty and diversity [28]. Uncertainty-based query strategies, such as least confidence, margin sampling, and entropy, select samples that are difficult to predict by the current model [29]. Diversity-based strategies select batches of unlabeled data samples that are representative of the unlabeled pool. This includes clustering algorithms such as the well-known KMeans algorithm [28] and selecting data samples from a small core set that tries to represent the full dataset distribution [30]. Lastly, hybrid strategies attempt to select samples that balance diversity and uncertainty. Example algorithms include BADGE [31], Exploitation–Exploration [32], and DBAL [33]. Zhan et al. [28] implemented 17 different query strategies for DAL and compared them across 7 datasets for image classification. They found unsatisfactory results for diversity-based strategies compared to uncertainty-based strategies and hybrid strategies. Based on their evaluation, they recommend trying uncertainty-based query strategies first for new tasks.

### 2.3. Deep Active Learning for Object Detection

While AL is traditionally used for classification tasks, the DAL cycle can also be used on OD tasks to reduce annotation costs. Because OD models can produce multiple detections per image, an aggregation method has to be used in order to compute a single score per image as input to the query strategy [34]. Brust et al. [35] trained a YOLO OD model [36] on the PASCAL VOC 2012 dataset [37] with DAL using margin sampling as an uncertainty-based query strategy. In their experimental evaluation, they compared the aggregation methods sum, maximum, and average to aggregate the uncertainty scores from multiple bounding box detections. They concluded that, overall, the sum was the best aggregation method for their data. Haussmann et al. [38] also compared different query strategies on a large-scale OD dataset including cars, pedestrians, bicycles, traffic signs, and traffic lights. As a model, they used a one-stage object detector based on a UNet [39]. They found that uncertainty-based query strategies and diversity-based strategies both performed better than random sampling. Furthermore, they found that letting the query strategy choose from a combined dataset consisting of the unlabeled pool *U* and the labeled set *L* outperforms *U* alone while reducing labeling costs.

As described in Section 2.2, before running the first DAL iteration, an initial model has to be trained. Usually, the initial model is trained by randomly selecting a first batch of samples as $L_{0}$ [28,35,38]. However, randomly sampling a small training set can lead to low initial model performance. Furthermore, randomly sampling a large initial training set increases the annotation cost, which is contrary to the goal of DAL. Therefore, in this work, we propose to train the initial model using synthetically generated images that include automatically generated annotations.

### 2.4. Combining Deep Active Learning with Synthetic Images

Peng et al. [40] combined synthetic images with DAL in surgical instrument segmentation. For each DAL cycle, they query the most informative training images according to the uncertainty-based query strategy Bayesian active learning by disagreement (BALD) [41] and then manually label them. Next, they generate additional synthetic images via copy-and-paste based on the selected images. The authors conclude that combining synthetic images with deep active learning for image segmentation results in improved performance, especially with limited labeled data. Similarly, query strategies are used in [42,43] to select a limited amount of relevant synthetic images to improve the available real training dataset. Wang et al. [44] combined AL and synthetic images for weakly-supervised OD. They generated synthetic training images via copy-and-paste from a few manually annotated images to train an initial base model. The synthetic images are used in the initial iteration, and weakly labeled images are used in subsequent iterations to train a teacher–student OD model.

Our proposed method uses available industrial 3D models to automatically generate training images via physics-based rendering for an initial OD model. During deployment, large amounts of unlabeled images can be collected. Given an unlabeled pool of images, DAL is used to efficiently fine-tune the next model iteration on a small number of manually labeled images.

## 3. Materials and Methods

**Figure 2:**
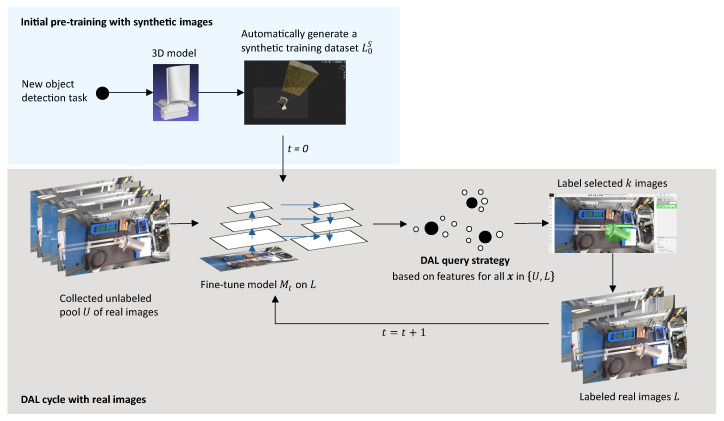
Proposed workflow to train and improve a data-efficient OD model throughout its life cycle.

The overall methodology of our approach is summarized in Figure 2. First, a synthetic training dataset $L_{0}^{S}$ is automatically generated according to Section 3.1, based on a given 3D model. With these synthetic images, an initial model $M_{0}^{S}$ is trained which can then be used for the first DAL cycle with a collected pool of unlabeled real images *U* (Section 3.2). The model chooses *k* real training images according to the DAL query strategy from Section 3.4. These images are labeled and added to the labeled training set *L*. Given the previous model and the selected training images, a new model is fine-tuned according to Section 3.3 and the DAL cycle can be repeated in the next iteration *t*.

### 3.1. Generating a Synthetic Training Dataset

The open-source 3D creation software Blender is a popular tool amongst many researchers to generate synthetic training images for computer vision tasks, e.g., [19,45,46,47]. Blender utilizes a path tracing rendering engine called Cycles for producing physically-based renders and can be automated using its Python API.

**Figure 3:**
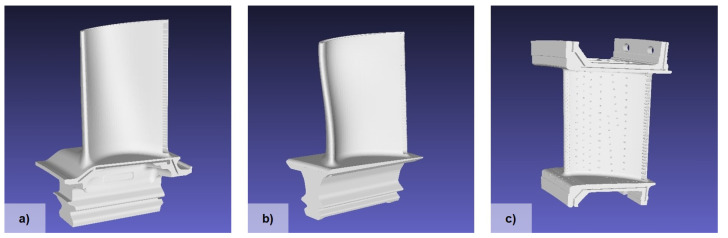
Three different industrial turbine blade models were used to generate synthetic training images. (**a**) Turbine blade 3D model 1. (**b**) Turbine blade 3D model 2. (**c**) Guide vane 3D model.

**Figure 4:**
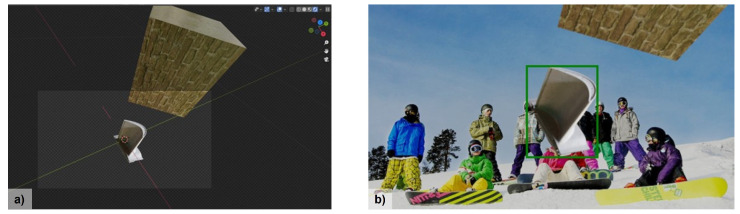
Synthetic data generation example. (**a**) Blender scene with a turbine blade and an additional distractor object. The box shows the camera view. (**b**) Generated image with bounding box annotation in green.

As described in more detail in our previous work [11], Blender v2.93 is used to automatically generate synthetic training images for a turbine blade detection task. In [11], various strategies for generating images were compared, including different lighting, background, object texture, additional foreground objects, and bounding box computation. Based on these results, a virtual camera is created for each scene and one of the three turbine blade models shown in Figure 3 is added with a randomized position. For the turbine blade models, a realistic-looking material texture is sampled from a pre-defined set of texture images that are either gray or dark blue. Furthermore, up to three distractor objects are added with a randomly selected material texture from a pool of texture images. For each virtual scene, a high dynamic range image is randomly sampled for image-based lighting. After rendering the scene, a random image from the COCO dataset [48] is added to the image background. Thus, we generate an automatically annotated synthetic training dataset consisting of 5000 different images for our generic turbine blade detection task. As an example, a Blender scene and the resulting annotated image are shown in Figure 4. Our code for generating synthetic training data based on 3D models is publicly available on GitHub (https://github.com/ignc-research/blender-gen, accessed on 28 December 2023).

### 3.2. Real Dataset of Our Industrial Object Detection Use Case

**Figure 5:**
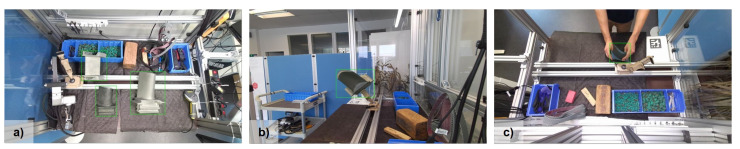
Annotated example images from the collected dataset. (**a**) Top view with three turbine blades on the table. (**b**) Side view with a clamped turbine blade. (**c**) Top view with a turbine blade in hand.

We collected 1300 images in 1080P quality from two Microsoft Azure Kinect cameras on an industrial workbench from our previous work [49] over several days. The images were collected from two different camera angles. Each image contains a minimum of one and a maximum of three turbine blades. Example images are depicted in Figure 5. Tools and additional objects on the workbench create a moderate amount of clutter. We randomly split the collected data into a pool of 1000 training images and 300 validation images.

### 3.3. Object Detection Model Training Details

For our object detection model, we used the Faster R-CNN [50] implementation from MMDetection [51], which uses a feature pyramid network [52] based on a ResNet-50 backbone [53] and is pre-trained on the Microsoft COCO dataset [48]. We trained all our models with stochastic gradient descent with an input image size of 640×360 , a batch size of 4, a learning rate of 0.00001 , a momentum factor of 0.9 , and a $L^{2}$ weight decay factor of 0.0001 [54]. To increase data efficiency, we use data augmentation during training. We used the library Albumentations [55] for online data augmentation, where we randomly performed flipping, color jitter, Gaussian noise, Gaussian blur, shifting, and scaling on training images. Augmenting training images is particularly useful when fine-tuning the model with small query batches of real images.

We trained all our models on an Nvidia GeForce RTX 3090 GPU until the average precision (AP) metric converged on the validation set. The AP metric is widely used to evaluate the performance of an object detection model. It computes the area under the precision-recall curve for a given threshold *T* and ranges from zero to one. Specifically, we use COCO’s *AP*@[0.5:0.95], which uses 10 different thresholds T=[0.5,0.55,⋯,0.95] regarding the bounding box intersection over union and averages them into one single metric. A mathematical definition of *AP*@[0.5:0.95] can be found in [56].

### 3.4. Deep Active Learning Pipeline

Based on the comparative survey of DAL query strategies from Zhan et al. [28], we implemented an uncertainty-based query strategy and a hybrid query strategy. For our experiments, a pre-trained model is needed to complete one DAL cycle. For experiments with real images only, a publicly available Faster R-CNN base model $M_{0}^{R}$ pre-trained on the COCO dataset was used. For experiments with synthetic images as described in Section 3.1, the COCO base model was fine-tuned on a labeled training set $L_{0}$ of 5000 synthetic images for 85 epochs, resulting in an average precision of *AP*@[0.5:0.95] = 0.555 for the synthetic base model $M_{0}^{S}$ .

#### 3.4.1. Uncertainty-Based Query Strategy

Considering the results from Brust et al. [35], we chose maximum margin sampling with the sum aggregation method as our uncertainty-based query strategy. In maximum margin sampling, an informativeness score $s_{margin}$ for a detected object $x_{d}$ is calculated according to Equation (1), where $P({\hat{y}}_{1}|x_{d})$ is the predicted probability of the class with the highest confidence and $P({\hat{y}}_{2}|x_{d})$ is the predicted probability of the second most confident class.

(1) $$s_{margin}\left(x_{d}\right)=1-\left[P\left({\hat{y}}_{1}|x_{d}\right)-P\left({\hat{y}}_{2}|x_{d}\right)\right]$$

Because an image *x* can contain *D* detections, an aggregation method is required to combine multiple detections into one score. The sum aggregation method $a_{sum}\left(x\right)$ simply computes the sum over all detections in an image according to Equation (2).

(2) $$a_{sum}\left(x\right)=\sum_{d\in D}^{\;}s_{margin}\left(x_{d}\right)$$

If the OD model returns zero detections for an image, then $a_{sum}\left(x\right)$ is set to zero. Intuitively, the uncertainty-based query strategy described in Algorithm 1 will select samples *x* with multiple uncertain detections per image.

**Algorithm 1** Maximum margin sampling**Input**: Unlabeled pool of images *U*, empty labeled training set *L*, query batch size *k*, pre-trained model 
$M_{0}^{S}$

**Output**: Fine-tuned model *M*
 1: 
t=1

 2: **loop**
 3:     Obtain informativeness score 
$a_{sum}\left(x\right)$
 for every image 
x∈{U,L}

 4:     **if** an image *x* has no detections **then**
 5:         Set 
$a_{sum}\left(x\right)=0$
 6:     **end if**
 7:     Select and label top *k* images with the highest scores, add them to *L* 8:     Fine-tune object detection model 
$M_{t}$
 on labeled training set *L* 9:     
t=t+1
 10: **end loop**


#### 3.4.2. Hybrid Query Strategy

As a hybrid query strategy, we chose the diverse mini-batch active learning (DBAL) algorithm from Zhdanov [33]. As described in Algorithm 2, DBAL first filters out training images with a low informativeness score by using a pre-filter factor β . To this end, the top βk images are selected for further processing. In our experiments, β=2 was used. Then, *k* diverse samples are selected from the remaining βk images with weighted KMeans++ clustering [57], where the weights are represented by the maximum margin informativeness scores. By selecting the image closest to each of the *k* clusters, the selected training images are expected to be more diverse.

In order to perform clustering, feature vectors that represent the training images *x* are required. We use the last feature map $P_{2}$ of size (256,90,160) from the feature pyramid network model $M_{0}^{S}$ [52] and perform global average pooling to convert the feature map to a one-dimensional feature vector of size 256. These feature vectors are then used for weighted KMeans++ clustering.

**Algorithm 2** DBAL**Input**: Unlabeled pool of images *U*, empty labeled training set *L*, query batch size *k*, pre-filter factor 
β
, pre-trained model 
$M_{0}^{S}$

**Output**: Fine-tuned model *M*
 1: 
t=1

 2: **loop** 3:     Obtain informativeness score 
$a_{sum}\left(x\right)$
 for every image 
x∈{U,L}
 4:     **if** an image *x* has no detections **then** 5:         Set 
$a_{sum}\left(x\right)=0$
 6:     **end if** 7:     Pre-filter to top 
βk
 informative images 8:     Cluster 
βk
 images to *k* clusters with weighted KMeans++ 9:     Select and label *k* images closest to the cluster centers, add them to *L* 10:     Fine-tune the object detection model 
$M_{t}$
 on labeled training set *L* 11:     
t=t+1
 12: **end loop**


## 4. Results

Using the described methodology from Section 3, we trained multiple OD models by combining synthetic data and DAL. As training data, we used either only real training images (R) or we used the synthetically pre-trained model $M_{0}^{S}$ and then fine-tuned it on real images (S+R). For DAL query strategies, we implemented the two described algorithms from Section 3.4.1 and Section 3.4.2. Additionally, we implemented a random sampling strategy as a baseline, which shuffles the unlabeled pool of images and then selects a batch of *k* training images randomly. We ran each random strategy three times using different random seeds.

### 4.1. Combining Synthetic Images and Deep Active Learning for One DAL Cycle

**Figure 6:**
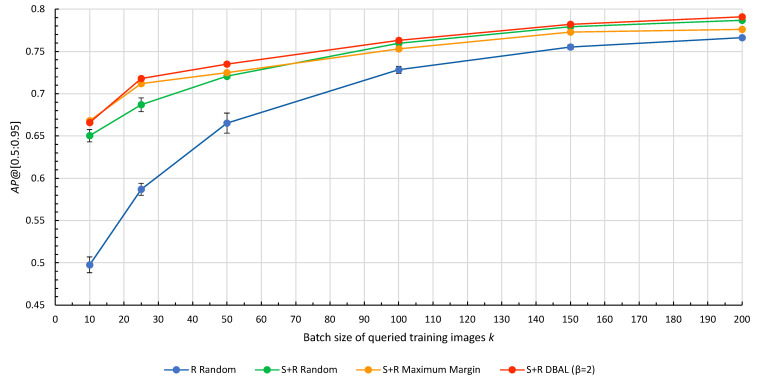
Results for the first DAL cycle with different query strategies. R Random: Baseline model using random sampling and only real images. S+R Random: Synthetic base model fine-tuned on real images with random sampling. S+R Maximum Margin: Synthetic base model fine-tuned on real images with Algorithm 1. S+R DBAL: Synthetic base model fine-tuned on real images with Algorithm 2.

First, we ran experiments for Algorithms 1 and 2, and random sampling for one DAL cycle with different query batch sizes *k*. Results for different DAL query strategies are shown in Figure 6. All numerical results can be found in the Appendix A in Table A1.

Using synthetic training images for model pre-training always outperformed using only real images. In fact, the difference between using synthetic images and not using synthetic images is much greater than the difference between the different query strategies. The results show that the importance of synthetic images increases as the number of labeled training images decreases. For k=10 , the model pre-trained on a synthetic dataset (S+R Random) increased the *AP*@[0.5:0.95] by 30.5% compared to the baseline model trained only on real images (R Random).

The hybrid query strategy DBAL has a higher AP than the random query strategy for all batch sizes *k* and shows overall the best performance. The chart shows that DAL query strategies are most useful with a small number of training images selected from a bigger pool of unlabeled data. The largest improvement over random sampling is at k=25 , where S+R DBAL increased the AP by 4.5% in comparison to S+R Random. In other words, using 25 real training images with S+R DBAL yielded equivalent AP results to randomly selecting about 50 training images. For large batch sizes with k⩾100 , neither DAL query strategy yielded a meaningful improvement in model performance over random sampling in the first DAL cycle. As *k* approaches the total number of images in *U*, all query strategies must converge eventually. As shown by the standard error, selecting training images randomly yields varying AP values due to dependence on the random seed. Therefore, employing DAL minimizes the chance of selecting an unfavorable random seed.

**Figure 7:**
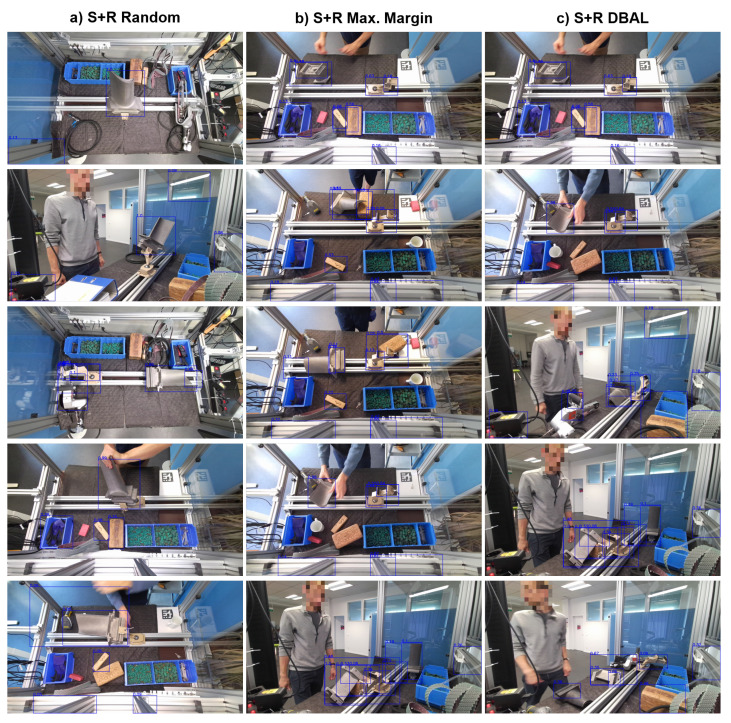
Top five training images for the initial model $M_{0}^{S}$ from the unlabeled pool *U* according to the different query strategies. Bounding box predictions are displayed in blue, including the turbine blade class confidence value. Best viewed with zoom. (**a**) Top five training images according to S+R Random. (**b**) Top five training images according to S+R Maximum Margin. (**c**) Top five training images according to S+R DBAL.

Figure 7 shows the top five selected images from the unlabeled pool *U* by the initial model $M_{0,S}$ according to the different query strategies in the first DAL cycle. As expected from Equations (1) and (2), maximum margin sampling and DBAL both select images from the unlabeled pool *U* with many false positive detections with high uncertainty.

### 4.2. Multiple Deep Active Learning Cycles

**Figure 8:**
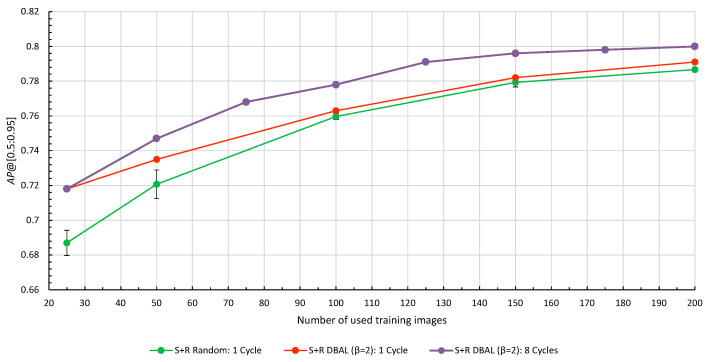
Results for one DAL cycle with varying batch sizes *k* compared to eight DAL cycles with a fixed batch size of k=25 .

Based on our findings in Section 4.1, we opted for DBAL as our query strategy with a fixed batch size of k=25 . Starting with the synthetic base model $M_{0}^{S}$ , the model was iteratively fine-tuned for eight DAL cycles according to Algorithm 2. At each cycle, the labeled training set *L* was extended by the 25 selected samples x∈{U,L} , based on the feature vectors from the previously trained model. Results for DBAL with up to t=8 DAL cycles are compared to the previous charts in Figure 8 for a single cycle. Numerical results can be found in the Appendix A in Table A1.

**Figure 9:**
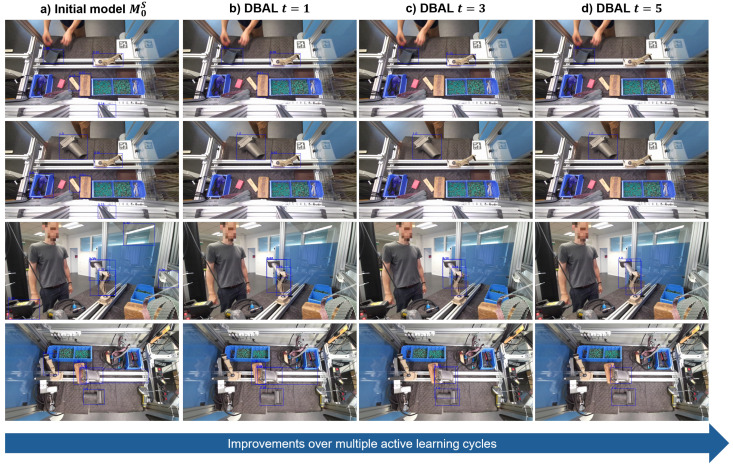
Qualitative results from S+R DBAL on validation images throughout multiple active learning cycles. Bounding box predictions are displayed in blue, including the turbine blade class confidence value. Best viewed with zoom. (**a**) Results from the initial model trained only on synthetic images. (**b**) Results after one cycle with real images. (**c**) Results after three cycles with real images. (**d**) Results after five cycles with real images.

The results presented in Figure 8 show that running DBAL for multiple DAL cycles yields better OD performance compared to running only a single cycle with an equivalent number of training images. For instance, a single cycle of DBAL with 150 labeled images performed the same as running four cycles of DBAL with 25 new images each time, which requires a maximum amount of 100 labeled images. Qualitative results on validation images are depicted in Figure 9 which shows the iterative learning of the model over the course of multiple DBAL cycles. False positive detections are reduced and the confidence values of turbine blade detections increase with each new cycle.

## 5. Discussion and Conclusions

To summarize, this work combined the generation of synthetic training images with DAL in order to train industrial OD models with minimal manual annotations. The base model is initially trained on automatically generated synthetic images and subsequently fine-tuned in each DAL cycle with real images. The synthetic base model enables early deployment, while unlabeled real training images can be collected over time. To ensure data efficiency, the DAL query strategy selects a limited batch of images for training from a larger pool of unlabeled images. On our turbine blade detection dataset, we found that using synthetic images for pre-training improved model performance, especially when the number of real training images was small. Additionally, the hybrid query strategy DBAL outperformed uncertainty-based maximum margin sampling and random sampling for small batch sizes. Furthermore, running multiple DAL cycles with a small batch size performed better than running only one cycle with an equivalent number of training images. Utilizing DAL can either increase model performance with the same amount of data, or provide the same performance with fewer data compared to randomly selecting training images. Additionally, employing DAL minimizes the risk of selecting an unfavorable batch of training images by chance.

Our findings are limited by our specific industrial use case of a turbine blade detection model. However, the presented methodology is not restricted to turbine blades and can be applied to any object. In future work, we plan to apply our approach to new industrial applications and datasets. For both of our implemented DAL query strategies, we used maximum margin as an informativeness score combined with the sum aggregation method. Choosing an alternative informativeness score and aggregation method could lead to different results. For our experiments with multiple DAL cycles in Section 4.2, we did not change the unlabeled pool of images *U*. However, during real-world deployment of an OD model, it is possible to collect new images over time. A steady increase in *U* will provide the DAL query strategy with a larger selection of images to choose from.

As a next step, we would like to train and iteratively improve multiple OD models using the developed workflow over a longer period of time on the shop floor. Future work should incorporate best practices from the machine learning operations (MLOps) paradigm [58] to automatically train and test new models and to ensure that each model update performs better than the previous model. Automatic triggering of a new DAL cycle could be initiated through continuous model monitoring. For instance, this could occur when a specific amount of new data in *U* are collected, a certain time period has passed, a dataset shift is detected [59], or model performance declines on key metrics.

## Data Availability

The industrial turbine blade data are not publicly available due to protection of intellectual property.

## Abbreviations

The following abbreviations are used in this manuscript:

AL	active learning
AP	average precision
DAL	deep active learning
DBAL	diverse mini-batch active learning
MLOps	machine learning operations
OD	object detection

## Appendix A

Numerical results from the experiments from Section 4 are documented in Table A1.

**Table A1:** Numerical results for all experiments. R: Only real training images were used; S+R: Synthetic base model fine-tuned with real training images.

Strategy	Number of Real Training Images	*AP*@[0.5:0.95] Random Seed 1	*AP*@[0.5:0.95] Random Seed 2	*AP*@[0.5:0.95] Random Seed 3	(Average) *AP*@[0.5:0.95]
R Random *	10	0.479	0.507	0.507	0.498
	25	0.578	0.582	0.601	0.587
	50	0.686	0.665	0.645	0.665
	100	0.733	0.732	0.720	0.728
	150	0.754	0.761	0.751	0.755
	200	0.765	0.771	0.763	0.766
S+R Random *	0				0.555
	10	0.636	0.655	0.660	0.650
	25	0.671	0.692	0.698	0.687
	50	0.724	0.718	0.720	0.721
	100	0.757	0.765	0.757	0.760
	150	0.778	0.782	0.778	0.779
	200	0.782	0.788	0.790	0.787
S+R Max. Margin	0				0.555
	10				0.668
	25				0.712
	50				0.725
	100				0.753
	150				0.773
	200				0.776
S+R DBAL	0				0.555
	10				0.666
	25				0.718
	50				0.735
	100				0.763
	150				0.782
	200				0.791
S+R DBAL (8 cycles)	0				0.555
	25				0.718
	50				0.747
	75				0.768
	100				0.778
	125				0.791
	150				0.796
	175				0.798
	200				0.800

* Random sampling strategies were repeated with three different random seeds.

## References

[1] Gupta C., Farahat A. Deep Learning for Industrial AI: Challenges, New Methods and Best Practices. Proceedings of the 26th ACM SIGKDD International Conference on Knowledge Discovery & Data Mining.

[2] Torralba A., Efros A.A. (2011). Unbiased look at dataset bias. Proceedings of the 2011 IEEE Conference on Computer Vision and Pattern Recognition (CVPR 2011).

[3] Coyner A.S., Chen J.S., Chang K., Singh P., Ostmo S., Chan R.V.P., Chiang M.F., Kalpathy-Cramer J., Campbell J.P. (2022). Synthetic Medical Images for Robust, Privacy-Preserving Training of Artificial Intelligence: Application to Retinopathy of Prematurity Diagnosis. Ophthalmol. Sci..

[4] Northcutt C., Athalye A., Mueller J., Vanschoren J., Yeung S. (2021). Pervasive Label Errors in Test Sets Destabilize Machine Learning Benchmarks. Proceedings of the Neural Information Processing Systems Track on Datasets and Benchmarks 1 (NeurIPS Datasets and Benchmarks 2021).

[5] Tobin J., Fong R., Ray A., Schneider J., Zaremba W., Abbeel P. (2017). Domain randomization for transferring deep neural networks from simulation to the real world. Proceedings of the 2017 IEEE/RSJ International Conference on Intelligent Robots and Systems (IROS).

[6] Lambrecht J., Kästner L. (2019). Towards the Usage of Synthetic Data for Marker-Less Pose Estimation of Articulated Robots in RGB Images. Proceedings of the 2019 19th International Conference on Advanced Robotics (ICAR).

[7] Nowruzi F.E., Kapoor P., Kolhatkar D., Hassanat F.A., Laganiere R., Rebut J. (2019). How much real data do we actually need: Analyzing object detection performance using synthetic and real data. arXiv.

[8] Movshovitz-Attias Y., Kanade T., Sheikh Y. (2016). How Useful Is Photo-Realistic Rendering for Visual Learning?. Lecture Notes in Computer Science.

[9] de Melo C.M., Rothrock B., Gurram P., Ulutan O., Manjunath B. Vision-Based Gesture Recognition in Human-Robot Teams Using Synthetic Data. Proceedings of the 2020 IEEE/RSJ International Conference on Intelligent Robots and Systems (IROS).

[10] Yang X., Fan X., Wang J., Lee K. (2022). Image Translation Based Synthetic Data Generation for Industrial Object Detection and Pose Estimation. IEEE Robot. Autom. Lett..

[11] Eversberg L., Lambrecht J. (2021). Generating Images with Physics-Based Rendering for an Industrial Object Detection Task: Realism versus Domain Randomization. Sensors.

[12] Schraml D. (2019). Physically based synthetic image generation for machine learning: A review of pertinent literature. Proceedings of the Photonics and Education in Measurement Science 2019.

[13] Georgakis G., Mousavian A., Berg A., Kosecka J. Synthesizing Training Data for Object Detection in Indoor Scenes. Proceedings of the Robotics: Science and Systems XIII. Robotics: Science and Systems Foundation.

[14] Dwibedi D., Misra I., Hebert M. (2017). Cut, Paste and Learn: Surprisingly Easy Synthesis for Instance Detection. Proceedings of the 2017 IEEE International Conference on Computer Vision (ICCV).

[15] Gorschlüter F., Rojtberg P., Pöllabauer T. (2022). A Survey of 6D Object Detection Based on 3D Models for Industrial Applications. J. Imaging.

[16] Tremblay J., Prakash A., Acuna D., Brophy M., Jampani V., Anil C., To T., Cameracci E., Boochoon S., Birchfield S. (2018). Training Deep Networks with Synthetic Data: Bridging the Reality Gap by Domain Randomization. Proceedings of the 2018 IEEE/CVF Conference on Computer Vision and Pattern Recognition Workshops (CVPRW).

[17] Prakash A., Boochoon S., Brophy M., Acuna D., Cameracci E., State G., Shapira O., Birchfield S. (2019). Structured Domain Randomization: Bridging the Reality Gap by Context-Aware Synthetic Data. Proceedings of the 2019 International Conference on Robotics and Automation (ICRA).

[18] Hodan T., Vineet V., Gal R., Shalev E., Hanzelka J., Connell T., Urbina P., Sinha S.N., Guenter B. (2019). Photorealistic Image Synthesis for Object Instance Detection. Proceedings of the 2019 IEEE International Conference on Image Processing (ICIP).

[19] Jabbar A., Farrawell L., Fountain J., Chalup S.K. (2017). Training Deep Neural Networks for Detecting Drinking Glasses Using Synthetic Images. Neural Information Processing.

[20] Pharr M., Jakob W., Humphreys G. (2016). Physically Based Rendering: From Theory to Implementation.

[21] Shrivastava A., Pfister T., Tuzel O., Susskind J., Wang W., Webb R. Learning From Simulated and Unsupervised Images Through Adversarial Training. Proceedings of the 2017 IEEE Conference on Computer Vision and Pattern Recognition (CVPR).

[22] Sankaranarayanan S., Balaji Y., Jain A., Lim S.N., Chellappa R. Learning From Synthetic Data: Addressing Domain Shift for Semantic Segmentation. Proceedings of the 2018 IEEE/CVF Conference on Computer Vision and Pattern Recognition.

[23] Peng X., Saenko K. (2018). Synthetic to Real Adaptation with Generative Correlation Alignment Networks. Proceedings of the 2018 IEEE Winter Conference on Applications of Computer Vision (WACV).

[24] Rojtberg P., Pollabauer T., Kuijper A. (2020). Style-transfer GANs for bridging the domain gap in synthetic pose estimator training. Proceedings of the 2020 IEEE International Conference on Artificial Intelligence and Virtual Reality (AIVR).

[25] Su Y., Rambach J., Pagani A., Stricker D. (2021). SynPo-Net—Accurate and Fast CNN-Based 6DoF Object Pose Estimation Using Synthetic Training. Sensors.

[26] Settles B. (2009). Active Learning Literature Survey.

[27] Ren P., Xiao Y., Chang X., Huang P.Y., Li Z., Gupta B.B., Chen X., Wang X. (2021). A Survey of Deep Active Learning. ACM Comput. Surv..

[28] Zhan X., Wang Q., hao Huang K., Xiong H., Dou D., Chan A.B. (2022). A Comparative Survey of Deep Active Learning. arXiv.

[29] Wang D., Shang Y. (2014). A new active labeling method for deep learning. Proceedings of the 2014 International Joint Conference on Neural Networks (IJCNN).

[30] Sener O., Savarese S. Active Learning for Convolutional Neural Networks: A Core-Set Approach. Proceedings of the 2018 International Conference on Learning Representations (ICLR).

[31] Ash J.T., Zhang C., Krishnamurthy A., Langford J., Agarwal A. Deep Batch Active Learning by Diverse, Uncertain Gradient Lower Bounds. Proceedings of the 2020 International Conference on Learning Representations (ICLR).

[32] Yin C., Qian B., Cao S., Li X., Wei J., Zheng Q., Davidson I. (2017). Deep Similarity-Based Batch Mode Active Learning with Exploration-Exploitation. Proceedings of the 2017 IEEE International Conference on Data Mining (ICDM).

[33] Zhdanov F. (2019). Diverse mini-batch Active Learning. arXiv.

[34] Li Y., Fan B., Zhang W., Ding W., Yin J. (2021). Deep active learning for object detection. Inf. Sci..

[35] Brust C.A., Käding C., Denzler J. (2019). Active Learning for Deep Object Detection. Proceedings of the 14th International Joint Conference on Computer Vision, Imaging and Computer Graphics Theory and Applications (VISIGRAPP).

[36] Redmon J., Divvala S., Girshick R., Farhadi A. You Only Look Once: Unified, Real-Time Object Detection. Proceedings of the IEEE Conference on Computer Vision and Pattern Recognition (CVPR).

[37] Everingham M., Gool L.V., Williams C.K.I., Winn J., Zisserman A. (2009). The Pascal Visual Object Classes (VOC) Challenge. Int. J. Comput. Vis..

[38] Haussmann E., Fenzi M., Chitta K., Ivanecky J., Xu H., Roy D., Mittel A., Koumchatzky N., Farabet C., Alvarez J.M. (2020). Scalable Active Learning for Object Detection. Proceedings of the 2020 IEEE Intelligent Vehicles Symposium (IV).

[39] Ronneberger O., Fischer P., Brox T. (2015). U-Net: Convolutional Networks for Biomedical Image Segmentation. Lecture Notes in Computer Science.

[40] Peng H., Lin S., King D., Su Y.H., Bly R.A., Moe K.S., Hannaford B. (2021). Reducing Annotating Load: Active Learning with Synthetic Images in Surgical Instrument Segmentation. arXiv.

[41] Houlsby N., Huszár F., Ghahramani Z., Lengyel M. (2011). Bayesian Active Learning for Classification and Preference Learning. arXiv.

[42] He H., Bai Y., Garcia E.A., Li S. ADASYN: Adaptive synthetic sampling approach for imbalanced learning. Proceedings of the 2008 IEEE International Joint Conference on Neural Networks (IEEE World Congress on Computational Intelligence).

[43] Niemeijer J., Mittal S., Brox T. Synthetic Dataset Acquisition for a Specific Target Domain. Proceedings of the IEEE/CVF International Conference on Computer Vision (ICCV) Workshops.

[44] Wang Y., Ilic V., Li J., Kisačanin B., Pavlovic V. ALWOD: Active Learning for Weakly-Supervised Object Detection. Proceedings of the IEEE/CVF International Conference on Computer Vision (ICCV).

[45] Denninger M., Sundermeyer M., Winkelbauer D., Olefir D., Hodan T., Zidan Y., Elbadrawy M., Knauer M., Katam H., Lodhi A. BlenderProc: Reducing the Reality Gap with Photorealistic Rendering. Proceedings of the Robotics: Science and Systems (RSS).

[46] Dirr J., Gebauer D., Yao J., Daub R. (2023). Automatic Image Generation Pipeline for Instance Segmentation of Deformable Linear Objects. Sensors.

[47] Druskinis V., Araya-Martinez J.M., Lambrecht J., Bøgh S., de Figueiredo R.P. A Hybrid Approach for Accurate 6D Pose Estimation of Textureless Objects From Monocular Images. Proceedings of the 2023 IEEE 28th International Conference on Emerging Technologies and Factory Automation (ETFA).

[48] Lin T.Y., Maire M., Belongie S., Hays J., Perona P., Ramanan D., Dollár P., Zitnick C.L. (2014). Microsoft COCO: Common Objects in Context. Computer Vision—ECCV 2014.

[49] Eversberg L., Lambrecht J. (2023). Evaluating digital work instructions with augmented reality versus paper-based documents for manual, object-specific repair tasks in a case study with experienced workers. Int. J. Adv. Manuf. Technol..

[50] Ren S., He K., Girshick R., Sun J. Faster R-CNN: Towards Real-Time Object Detection with Region Proposal Networks. Proceedings of the Advances in Neural Information Processing Systems.

[51] Chen K., Wang J., Pang J., Cao Y., Xiong Y., Li X., Sun S., Feng W., Liu Z., Xu J. (2019). MMDetection: Open MMLab Detection Toolbox and Benchmark. arXiv.

[52] Lin T.Y., Dollár P., Girshick R., He K., Hariharan B., Belongie S. Feature Pyramid Networks for Object Detection. Proceedings of the 2017 IEEE Conference on Computer Vision and Pattern Recognition (CVPR).

[53] He K., Zhang X., Ren S., Sun J. Deep Residual Learning for Image Recognition. Proceedings of the 2016 IEEE Conference on Computer Vision and Pattern Recognition (CVPR).

[54] Goodfellow I., Bengio Y., Courville A. (2016). Deep Learning.

[55] Buslaev A., Iglovikov V.I., Khvedchenya E., Parinov A., Druzhinin M., Kalinin A.A. (2020). Albumentations: Fast and Flexible Image Augmentations. Information.

[56] Padilla R., Passos W.L., Dias T.L.B., Netto S.L., da Silva E.A.B. (2021). A Comparative Analysis of Object Detection Metrics with a Companion Open-Source Toolkit. Electronics.

[57] Arthur D., Vassilvitskii S. k-means++: The Advantages of Careful Seeding. Proceedings of the Eighteenth annual ACM-SIAM symposium on Discrete algorithms.

[58] Kreuzberger D., Kühl N., Hirschl S. (2023). Machine Learning Operations (MLOps): Overview, Definition, and Architecture. IEEE Access.

[59] Moreno-Torres J.G., Raeder T., Alaiz-Rodríguez R., Chawla N.V., Herrera F. (2012). A unifying view on dataset shift in classification. Pattern Recognit..

